# Supplementary material for: Chromothripsis during telomere crisis is independent of NHEJ, and consistent with a replicative origin
Source: Genome Res. 2019 May;29(5):737–49. doi: 10.1101/gr.240705.118 (PMC6499312; doi:10.1101/gr.240705.118)
Supplement: Supplemental Material [file supp_gr.240705.118_Supplemental_file_1.zip › contigs/annotated_contigs/DB50/contig.5.DB50_length_832_mean_cov_3.859375.docx]

**DB50_length_832_mean_cov_3.859375**

```
ATAGCCTTAATTGAAATCTTCATAAAATGCATTCTTTGAATAACAGCATC|AATTTT|TAAAAAGCAAAATAACAACAATGACAAAAAG
 >chr10:8954743-8954799 - E=8e-22 p=1e-02          >chr10:8025831-8026024 + E=3e-104
AATTTTAAGAATGACTGGTGAAGTTCACAAGTGACATTTAACATTTTTAAATGACTGTATTTAAAATTTTCAAATGACCTATTTTTAAT

GCTAGGGAAAGATGCTAACATTACCAGTGAAATTCCTAGGCTCTGAACAACTGCAACAAAGGTTG|TG|CTGGATGCAGCAAGGGTTGG
                                                                  >chr10:8163780-816417
GCGTGGTGGCTCACACCTGCAATCCCAGCACTTTGGGAGGCTGAGGTGGGAGGATTGCTGGAGCCCAGGAGTTCAAGACCAGCCTGGGC
4 - E=4e-225
AACATAGCAAGACCCCATCTCCACACACACAAAAAAATATCCAGCAGTAGTGGTGTGCACATGTAATCCTAGATACTCGGGAGGCTGAG

ACAGGAGGATAGCTTGAGCCCAGGAGTAGTTTAAGGCTCCAATGAGATCAGATTACACCACTGCACTCCAGCCTGGGTGACAGAGACCT

TGTCTCTAAAAAATAAAAATAAATCTCCATTCCAGATCTGAGGGCAGGAGGAAGTTTGGGGAAGGCTTTCCTGAAAAGGTGACAAGTGA

ATCAGGTGAAGGGGTC|TTCTTCATT|CCTGAGTTTCCTGGCCATGAGGCACACCTCTTTCACTGCA|CG|TGTTAGCTAGAGGTTGAA
                          >chr10:27600105-27600145 + E=3e-12        >chr10:8016683-80
GAGAAGTTAAGAAAATGTTTAAGATGGAGTGGAGCACATTTAAAAGTGAGATGAAGGCCGGGCACAGTGGCTCACACCCGTAATCCCAG
16829 + E=6e-76
CACTTTGGGAGGTCGAGGCGGGCGGATCACGAGGTCAGG
```
